# What Is the Role of Interleukins in Breast Cancer Bone Metastases? A Systematic Review of Preclinical and Clinical Evidence

**DOI:** 10.3390/cancers11122018

**Published:** 2019-12-13

**Authors:** Francesca Salamanna, Veronica Borsari, Deyanira Contartese, Viviana Costa, Gianluca Giavaresi, Milena Fini

**Affiliations:** 1 Laboratory Preclinical and Surgical Studies, IRCCS Istituto Ortopedico Rizzoli, 40136 Bologna, Italy; francesca.salamanna@ior.it (F.S.); deyanira.contartese@ior.it (D.C.); gianluca.giavaresi@ior.it (G.G.); milena.fini@ior.it (M.F.); 2 Innovative Technological Platforms for Tissue Engineering, Theranostic and Oncology, IRCCS Istituto Ortopedico Rizzoli, 90133 Palermo, Italy; viviana.costa@ior.it

**Keywords:** bone metastases, breast cancer, interleukins, preclinical studies, clinical trials

## Abstract

Breast cancer cells produce stimulators of bone resorption known as interleukins (ILs). However, data on the functional roles of ILs in the homing of metastatic breast cancer to bone are still fragmented. A systematic search was carried out in three databases (PubMed, Scopus, Web of Science Core Collection) to identify preclinical reports, and in three clinical registers (ClinicalTrials.gov, World Health Organization (WHO) International Clinical Trials Registry Platform, European Union (EU) Clinical Trials Register) to identify clinical trials, from 2008 to 2019. Sixty-seven preclinical studies and 11 clinical trials were recognized as eligible. Although preclinical studies identified specific key ILs which promote breast cancer bone metastases, which have pro-metastatic effects (e.g., IL-6, IL-8, IL-1β, IL-11), and whose inhibition also shows potential preclinical therapeutic effects, the clinical trials focused principally on ILs (IL-2 and IL-12), which have an anti-metastatic effect and a potential to generate a localized and systemic antitumor response. However, these clinical trials are yet to post any results or conclusions. This inconsistency indicates that further studies are necessary to further develop the understanding of cellular and molecular relations, as well as signaling pathways, both up- and downstream of ILs, which could represent a novel strategy to treat tumors that are resistant to standard care therapies for patients affected by breast cancer bone disease.

## 1. Introduction

Breast cancer represents the most recurrent malignancy among women, contributing to over 25% of the total number of new diagnosed cancers, and the second most frequent cause of cancer death [1,2]. Between 20% and 30% of patients with breast cancer develop metastases [3], with bone metastases occurring in about 15% of these patients [4]; about 50% of metastases involve bone as the primary metastatic site, while 80% involve bone as a secondary and/or recurring site [5,6,7]. Breast cancer bone metastatic patients have an increased morbidity, mainly due to hypercalcemia, bone fractures, spinal cord compression, impaired mobility, and pain [8]. This last factor is commonly caused by the mechanical pressure carried out by the tumor mass and/or by the release of inflammatory cytokines from tumor cells and/or from the bone microenvironment, thus causing an alteration of the bone homeostasis. All these observations emphasize the importance of understanding the mechanism(s) through which breast cancer cells grow and colonize the bone. Variances in anatomical sites and clinical pathology, together with the heterogeneity of molecular and cellular factors, make it difficult to predict susceptibility to bone metastases.

Breast cancer bone metastasis is mainly an osteolytic disease, but osteoblastic and mixed lesions can also occur [9,10,11]. During osteolytic bone metastases, osteoclast activity and/or the inhibition of osteoblast differentiation are stimulated by tumor cells, bringing an increase in bone resorption and/or a decrease in bone formation, respectively, leading to bone disease and higher risk of fractures [9,10,11]. As already well documented, the tumor microenvironment plays a central role in breast cancer progression. Alteration in the crosstalk between bone cells (osteoblasts or osteoclasts) and tumor cells promotes the release of secreted factors responsible for pre-metastatic niche formation [9,12,13,14,15,16]. In this complex network of molecules and factors, interleukins (ILs) were identified as key actors able to act on bone homeostasis and tumor cells stimulating tumor growth and bone destruction [12,17]. ILs are a family of small proteins implicated in cell signaling, originally isolated from leukocytes; however, it is now known that ILs are produced by nearly all cell types including osteoclasts, osteoblasts, osteocytes, and mesenchymal stem cells. ILs are modulators of immune response and are implicated in the regulation of cell functions, activation, differentiation, and proliferation, as well as cell–cell communication. Thus, it is evident that ILs can have a pivotal role in bone metastasis development. They can also be produced by tumor cells with a variety of functions, and they may act as tumor-promoting or tumor-inhibiting factors. To date, numerous ILs were investigated; however, available literature data do not provide a complete overview of IL role(s), function(s), and mechanism(s) of action in breast cancer bone metastasis pathogenesis and therapy. Having a complete framework of these aspects would provide specific indication of the role of ILs in breast cancer bone metastasis, and it could give specific evidence for the evaluation of new potential therapeutic IL-targeted approaches. Thus, we performed a systematic review of the latest evidence on the roles and functions of ILs that act in the breast cancer bone metastatic process. Finally, we also evaluated IL-based therapies that could be employed for the treatment of breast cancer bone metastases.

## 2. Method

### Literature Search

A systematic search of preclinical articles published in English between May 2008 and October 2019 was conducted in three electronic databases, PubMed, Scopus, and Web of Science Core Collection, using the keywords (interleukin OR interleukins) AND (breast cancer bone metastasis OR breast cancer to bone OR breast cancer in bone OR breast cancer skeletal metastasis OR metastatic breast cancer to bone). Subsequently, a public reference manager (Mendeley 1.17.11, Mendeley Ltd, London, UK) was used to eliminate duplicates. Two reviewers screened titles and abstracts, in addition to the full text, against the pre-specified criteria. 

In order to evaluate current or concluded clinical trials, some of the major clinical registry websites were also checked (ClinicalTrials.gov, World Health Organization (WHO) International Clinical Trials Registry Platform, European Union (EU) Clinical Trials Register). The search was applied with the following string: “(breast cancer) and (interleukin)”, “(breast cancer), and (anti-IL)”.

**Figure 1:**
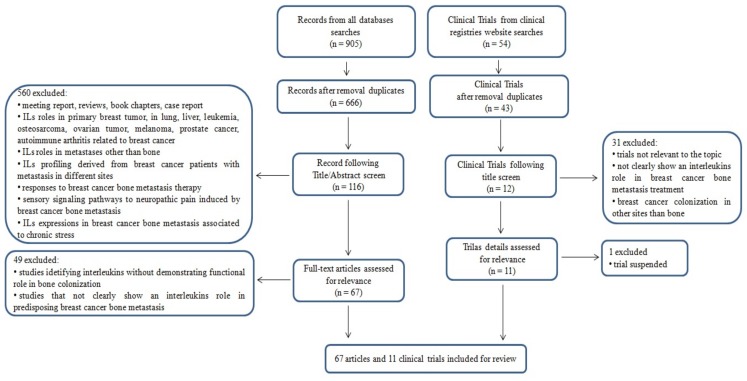
Preferred Reporting Items for Systematic Reviews and Meta-Analyses (PRISMA) flow chart of search criteria.

Detailed inclusion and exclusion criteria are reported in the Preferred Reporting Items for Systematic Reviews and Meta-Analyses (PRISMA) flow chart shown in Figure 1. This article does not contain any study performed by any of the authors with human participants or animals.

## 3. Results

### 3.1. Literature Search

An initial literature search found 905 references, of which 151 articles were recognized using the PubMed database, 481 articles were recognized using Scopus, and 273 articles were found in the Web of Science Core Collection. Subsequently, the resulting references were submitted to a public reference manager (Mendeley 1.17.11) to eliminate duplicate articles, obtaining 666 articles. From the 666 articles obtained, after exclusion, 116 full-text articles were evaluated, of which 67 met the inclusion criteria. The 560 studies excluded from this review were excluded because they were meeting reports, reviews, book chapters, and case reports, and/or because they reported IL roles in primary breast tumor, as well as in lung, liver, and pulmonary metastasis, leukemia, osteosarcoma, ovarian tumor, melanoma, prostate cancer, autoimmune arthritis related to breast cancer, and other pathological conditions; however, they did not reveal the favorable development of bone metastases from breast cancer. In addition, we excluded studies where IL profiling was derived from breast cancer metastatic patients that were not grouped/analyzed based on the site of metastasis, and where breast cancer bone metastases were evaluated, but no IL roles/functions were examined. Finally, we also eliminated studies where responses to specific therapy (not IL-based) for breast cancer bone metastasis were analyzed, studies that evaluated the contributions of the sensory signaling pathways to neuropathic pain induced by breast cancer bone metastases, and studies on chronic stress and IL expression in breast cancer bone metastasis (Figure 1).

Fifty-four clinical trials were found in the clinical registry websites (ClinicalTrials.gov, WHO International Clinical Trials Registry Platform, EU Clinical Trials Register). The results from registries were collected, assessed for relevance, and matched to eliminate any duplicates. After the screening process, 11 clinical trials were selected that focused on the topic of the present review.

Data were extracted to associate, arrange, compare, and combine the results. The retrieved data for preclinical studies (in vitro and in vivo) were as follows: author and publication date, interleukin(s) identified, interleukin(s) source, aims, and experimental approaches (Table 1). Extracted data for clinical trials were as follows: trial number of registries, aim, arms of the study, identified ILs and dosage, IL function, and summary of main findings (Table 2).

### 3.2. Findings from Preclinical Studies

As reported in Table 1 and references therein, in the 67 preclinical articles extracted in this review, several ILs, i.e., IL-1β, IL-2, IL-6, IL-8, IL-10, IL-11, IL-15, IL-17, IL-18, and IL-20 were linked with breast cancer cells homing to bone. Forty-one studies (61%) were in vitro and, of them, 33 used established human (MDA-MB-231, MDA-231B, T-47D, ZR-75-1, 435/BRMS1, 231/BRMS1, MDA-MB-435, MCF-7, BT474, SKBR-3, and Hs578T) or murine (MRMT-1, 13762 MAT B III, 4T1, 4T1.2, and 67NR) breast cancer cells lines, normal or modified (i.e., transfected), cultured alone and/or in direct or indirect co-culture with bone cells (osteoblasts, osteoclasts, fibroblasts, bone marrow-derived cells) and/or bone fragments, while the remaining studies (*n* = 8) used peripheral blood and tissue from breast cancer bone metastatic patients. Twenty-six studies (39%) were in vivo or both in vitro and in vivo, and they used intracardiac, intratibial, and subcutaneous injection of breast cancer cell lines (MDA-MB-231 variants, 4T1, 4T1.2, MDA-P, MDA-MET, NT2.5, MCF-7), normal or transfected, into mice or rats. 

**Figure 2:**
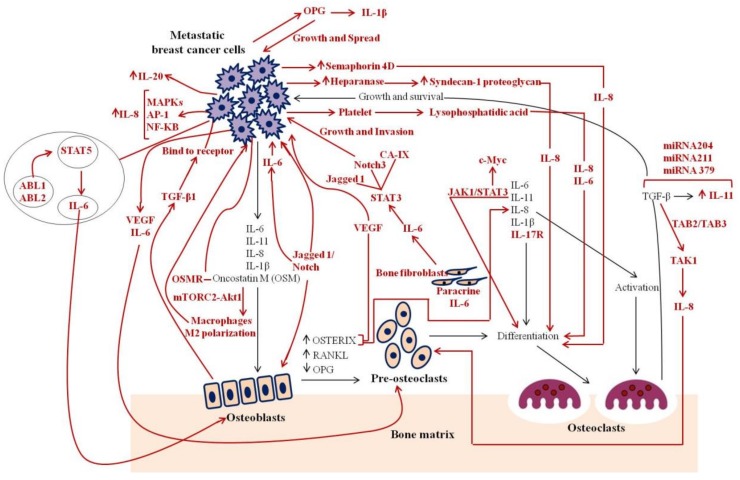
Mechanisms that regulate the interactions between breast cancer cells and bone. Black lines indicate established interactions of interleukins (ILs) within the vicious cycle. Red lines indicate potential additional interactions reviewed in this paper [18,19,20,21,22,23,24,25,26,27,28,29,30,31,32,33,34,35,36,37,38].

The examined papers mainly focused on (1) evaluation of the upregulation or downregulation of the expression of ILs during breast cancer bone metastases, (2) inhibition, blockade, and/or neutralization of Is signaling, by using IL dual-selective antagonists, anti-IL, anti-IL receptor, and IL monoclonal antibodies (mAb) in breast cancer bone metastases, and (3) definition of the role of ILs as potential biomarkers during breast cancer bone metastases. Although focused on different IL functions and roles in breast cancer bone metastases, almost all the examined studies supported the vicious cycle of breast cancer metastasis to bone that is driven by four main contributors: tumor cells, bone-forming osteoblasts, bone-destroying osteoclasts, and the organic bone matrix. However, this is an oversimplification of the breast cancer bone metastasis mechanism, and a more complex crosstalk between cells, cytokines, and growth factors is present. In fact, in this review, several studies (*n* = 21, 31%) evaluated new and unexplored mechanisms of action mediated by ILs in breast cancer bone metastasis. Findings of these mechanisms are schematically illustrated in Figure 2 and detailed in the subsequent paragraphs.

#### 3.2.1. IL-1

IL-1, a prototypic pro-inflammatory cytokine that presents itself in two forms, i.e., IL-1α and IL-Iβ, seems to be involved in different molecular mechanisms underlying primary breast cancer development and the formation of metastasis in bone. IL-1β involvement in breast cancer bone metastases was highlighted by its high expression in metastatic breast cancer cell lines, in serum from mice-bearing bone metastatic tumors and also in tissue samples from patients with breast cancer bone metastases [39,40]. Increased levels of IL-1β were also detected using three-dimensional (3D) in vitro models of breast cancer bone metastases where different breast cancer cell lines were cultured with bone tissue fragments from non-osteoporotic [41,42] and osteoporotic patients [43]; this last study also showed a higher expression of IL-1β in comparison to non-osteoporotic patients [43]. Increased IL-1β levels in a 3D model of breast cancer bone metastases were also associated with increased expression of adipokine/cytokine leptin, underling not only the critical role of IL-1β in the breast cancer bone metastatic niche but also in bone marrow adipose tissue [41]. A positive correlation between IL-1β expression and osteoprotegerin (OPG) was also found, revealing a potential role for OPG in the invasion-promoting effects of IL-1β and showing that IL-1β led to an increase in OPG production, via the p38 and p42/22 mitogen-activated protein kinase (MAPK) signaling pathway, independent of breast cancer cell subtype [18,19]. Since breast cancer cells express elevated levels of not only IL-1β but also other pro-inflammatory cytokines, Safina et al. showed, using an in vitro model showed that IL1-β and TNF-α cooperate with TGF-β in the production of MMP-9 by breast cancer cells and TGF-β activated protein kinase 1 (TAK1) is required for this process [30]. Additionally, co-culturing breast cancer cells with mice osteoblasts, it was seen that breast cancer cells were attached to the matrix, produced by osteoblasts, but grew slowly or not at all until TNF-α and IL-β addition [44]. Stimulation of cell proliferation by these cytokines was suppressed with indomethacin, an inhibitor of cyclooxygenase and of prostaglandin production, or a PGE2 receptor antagonist, showing that IL-1β and TNFα activate the arachidonic acid pathway [44]. Since the above mentioned studies underlined that IL-1β directly or through specific molecular mechanisms impacts on tumor aggressiveness and bone metastatic potential, several studies hypothesized that the blocking of IL-1β activity should have the potential to be an effective anti-cancer therapy. In fact, blocking of IL-1R signaling with the clinically licensed antagonist, i.e. anakinra, before injection of tumor cells in mice inhibited the development of metastases [45]. When the IL-1β dual selective antagonist was injected 1 week after tumour cell inoculation the existing bone metastases stopped growing [45]. Additionally, it was observed that in tumour-naïve mice, a single dose of IL-1β dual selective antagonist reduces osteoclast and osteoblast activity and IL-1β expression, also following continuous administration of IL-1β dual selective antagonist for more than 21 days [45]. Similarly, Tulotta et al. [46] using patient samples with stage II/III breast cancer, humanized mouse models of spontaneous breast cancer bone metastasis, genetic manipulation of breast cancer cells (MDA-MB-231-IL-1B+, T47D-IL-1B+ and MCF7-IL-1B+) and in vitro models (co-culture between HS5 or OB1 cells and MDA-MB-231 or T47D cells) demonstrated that not only the IL-1R antagonist, anakinra, but also the anti that a IL-1β antibody, canakinumab, inhibited breast tumor growth and progression to bone metastasis. Additionally, the production of IL-1β by tumor cells promoted epithelial–mesenchymal transition, invasion, migration, and bone colonization. Contact between tumor and osteoblasts or bone marrow cells increased IL-1β secretion from all three cell types [46].

Finally, only one study evaluated IL-1α expression using an in vitro model where breast cancer cells were treated with an endothelin-1 (ET-1) receptor dual antagonist, showing a local increase in breast cancer cell secretions of IL-1α [47].

#### 3.2.2. IL-2

IL-2, a master activation factor for helper/regulatory T-cell and natural killer (NK) cell proliferation and differentiation, acting as a mediator for pro- and anti-inflammatory immune responses, is also associated with breast cancer bone metastases. According to the paper of Iakovou et al., who assessed the levels of serum cytokines in women with bone metastases treated with radionuclide palliative therapy (RTP), responders to therapy showed higher levels of IL-2, a cytokine released by T-helper leucocytes, which have a role in the antitumor immune response, but no prognostic value [48].

#### 3.2.3. IL-6

IL-6, a multifunctional cytokine that was originally characterized as acting in immune and inflammatory responses, appears to play a key role in breast cancer growth and bone metastasis. Its role in breast cancer growth and bone metastasis was confirmed by evaluating blood samples from healthy, breast cancer, or metastatic breast cancer patients, where the higher levels of IL-6 were detected in patients with metastatic bone disease [49,50]. This higher expression of IL-6 in blood samples from patients with metastatic bone disease also correlated with high levels of Y-box-binding protein 1 expression, a multifunctional cold-shock protein [51], but not with cystatin C (Cyst C), an endogenous inhibitor of cysteine proteinase cathepsin K [52]. Increased levels of IL-6 were also highlighted in sera of mice with bone metastases [40,53], and by using a humanized 3D breast cancer bone metastasis model in which the higher levels of IL-6 were found in the presence of an osteoporotic microenvironment [43]. However, in order to evaluate the main contributors involved in IL-6 expression during breast cancer bone metastases, numerous in vitro and in vivo studies analyzed tumor cells, bone cells, immune cells, and the organic bone matrix. Several preclinical studies showed that metastatic breast cancer cells, directly [54,55,56,57,58,59] or through Jagged1-expressing tumor cells [58], induced osteoblasts to express high levels of IL-6 [54,55,56,57,58,59,60,61], while they seemed to not affect IL-6 production by osteoclasts [62,63]. The high expression of IL-6 by osteoblasts, particularly activated throughout the bone marrow [60], completely suppresses osteoblast functions [55] and stimulates osteoclastogenesis in the presence or absence of the receptor activator of nuclear factor kappa-Β (RANK)/RANK ligand (RANKL) pathway [56,59]. IL-6 induces the expression of RANK by breast cancer cells, which sensitizes the tumor to RANKL and enhances cancer IL-6 release [64]. RANKL and IL-6 mediate direct paracrine/autocrine signaling between cells of the osteoblast lineage and cancer cells, enhancing the growth of metastatic breast cancers within bone [59,64]. The disruption of this crosstalk by knockdown of IL-6 or RANK in breast cancer cells, or via treatment with anti-IL-6 receptor antibodies or an antagonist molecule, i.e., TB-2-081, significantly reduced breast cancer growth in bone [27,28,64,65], as well as in the presence of hormonal therapy (HT)-resistant metastatic disease where the anti-IL-6 receptor would block the IL-6^hi^/estrogen (ER)^lo^ feed-forward loop [26]. Additionally, the blockade of IL-6 induced with TB-2-081 increased phosphorylation of the signal transducer and activator of transcription 3 (pSTAT3) in breast cancer cells and reduced osteolytic bone remodeling [28]. In fact, it was observed that STAT3-dependent upregulation of Notch-3, Jagged-1, and carbonic anhydrase IX correlates with growth and invasion of breast cancer cells to bone [25]. Neutralization of IL-6 was also sufficient to limit senescent-induced reactive osteoblasts which are responsible for the increased osteoclastogenesis via increased IL-6 production [66]. Moreover, the in vivo knockdown of oncostatin M (OSM), a pleiotropic IL-6 family cytokine, seems to decrease breast cancer bone metastasis [35,38]. OSM also induced osteoclast differentiation via an amphiregulin, an uncharacterized OSM-regulated bone metastasis factor, through an autocrine loop [35]. In addition, OSM skews macrophages toward an M2 polarized phenotype via the mTOR (mammalian target of rapamycin) signaling complex 2 (mTORC2) which relays signals through PKC-α (protein kinase C-α) and Akt (protein kinase B) kinases [38]. However, in addition to the effect of IL-6 antagonists, anti-IL-6 receptors, and knockdown of OSM on IL-6 expression, a reduced level of IL-6 was also observed in blood samples of women with breast cancer bone metastases treated with radionuclide palliative therapy (RPT) [48] and in breast cancer cell lines treated with the miR-520/373 family [24], or where the ABL (Abelson murine leukemia) family of non-receptor tyrosine kinases, ABL1 (also known as c-Abl) and ABL2 (also known as Arg), was depleted [36]. The decrease in IL-6 secretion by depleting ABL kinases was accompanied by an enhanced OPG expression and reduced overall RANKL/OPG ratio, thereby decreasing osteoclast differentiation and bone destruction [36]. Other potential targets for bone metastatic breast cancer could be the calcium sensing receptor (CaSR) and the bioactive lipids lysophosphatidic acid (LPA) and sphingosine 1-phospahte (S1P) [27,34]. The role of CaSR as an inhibitor of the constitutive secretion of various cytokines was detected both in cancer cells and in normal breast cells. In breast cancer cells, CaSR inhibited IL-6 secretion [27], while LPA and S1P enhanced IL-6 expression [34]. On the other hand, based on the key role of low-molecular-weight protein tyrosine phosphatase slow isoform (LMW-PTPsi) in the interplay between tumor cells and osteoclasts during bone metastases, it was seen that the knockdown of LMW-PTP and its slow isoform did not decrease IL-6 expression in breast cancer cells [67].

#### 3.2.4. IL-8

IL-8, a prototypical member of a superfamily of small, inducible, secreted CXCs (chemokines) or α-chemokines originally identified as monocyte-derived factors capable of attracting and activating neutrophils, is expressed by a number of cancer cell lines in vitro. Numerous correlations were observed between breast cancer tumor cell IL-8 expression, plasma levels of IL-8, and metastatic potential to bone [54,68,69], also evaluating how chemical and physical properties of specific biomineralized culture platforms can alter breast cancer cell growth and secretion of tumorigenic IL-8 [70]. Increased levels of IL-8 were also highlighted in sera of mice with bone metastases and, together with IL-6, it might be responsible for the attraction of cancer stem-like cells to bone and might support the phenotypic switch [53]. Also, by using a humanized breast cancer bone metastasis model, increased levels of IL-8 were seen, in particular under osteoporotic conditions [43]. Metastatic breast cancer cells directly induce osteoblasts to express increased levels of IL-8 [54,56,68,71,72,73], especially under osteoporotic conditions [71], in the presence or in absence of the RANK/RANKL pathway [56]. Osteoblasts release several growth factors and, among them, TGF-β1 (Transforming Growth Factor – β1) is an important contributor to extracellular signal-related kinase (ERK), p38, and c-Jun N-terminal kinase (JNK) activation [72], which lead to the activation of activator protein 1(AP-1) and nuclear factor kappa-light-chain-enhancer of activated B cells (NF-κB) on the IL-8 promoter, and which initiate IL-8 release, thus promoting breast cancer cell migration and osteoclastogenesis [21,54,69,72]. In addition, IL-8 and other pro-inflammatory cytokines also cooperate with TGF-β1 in the production of matrix metalloproteinase-9 (MMP-9) by breast cancer cells, and TGF-β-activated protein kinase 1 is required for this response, contributing to the metastatic potential of breast cancer cells [30]. By using measurements of osteoblast and osteoclast differentiation and function in vitro and a mouse model of skeletal metastasis, it was demonstrated that both soluble Sema4D (semaphoring 4D)and protein, an immune semaphore expressed by T-lymphocytes and eosinophils and to a lesser extent by dendritic cells and B-lymphocytes, produced by the breast cancer cell line, inhibit the differentiation of osteoblast cells, while Sema4D-mediated induction of IL-8 and LIX/CXCL5 (C-X-C motif chemokine 5), the murine homologue of IL-8, increases osteoclast numbers and activity [37]. Using in vitro assays of osteoclastogenesis and bone resorption, it was shown that enhanced osteoclastogenesis also requires the presence of syndecan-1, a proteoglycan whose shedding from tumor cell surfaces is enhanced by the expression of heparanase and protein agents, such as IL-8 [31]. Others showed that breast cancer cells can induce the release of lysophosphatidic acid (LPA) from activated platelets which, in turn, promotes tumor cell proliferation and the LPA-dependent secretion of IL-8, thereby also enhancing tumor growth and osteolysis [33,34]. Despite several factors, proteins, and mechanisms that lead to high expression of IL-8, it was also seen that metastatic breast cancer cells treated with the miR-520/373 family [24] or knockdown for LMW-PTP and its slow isoform [67] showed a decrease level of IL-8 expression in breast cancer cells [24,67]. In addition, similar results were observed in vivo using a nude mice model injected into the tibia with metastatic breast cancer cells and treated via neutralizing IL-8 [52] or knockdown of osterix [29], a zinc finger-containing transcription factor essential for osteoblast differentiation and bone formation, suggesting a novel and attractive target for the control of bone metastasis of breast cancers [29].

#### 3.2.5. IL-10

Salamanna et al. found that osteoporosis modulates the release of cytokines as the anti-inflammatory mediator IL-10, in a 3D in vitro model where bone samples were cultivated with MCF-7 [43].

#### 3.2.6. IL-11

IL-11, a member of the IL-6 family, is an osteolytic factor produced by breast cancer cells, and its expression seems to be associated with the development of bone metastases. Mendoza et al. showed that IL-11 expression is regulated by runt-related transcription factor 2 (Runx2) in human breast cancer cells [74]. Induction of IL-11 in metastatic breast cancer cells is controlled by TGF-β in a dose-dependent manner [75] through different signaling pathways, such as SMAD (small mother against decapentaplegic) and p38 MAPK [21]. TGF-β-induced IL-11 expression can also be regulated by specific microRNAs (miRNAs). Pollari et al. [22,23] identified miR-204, -211, and -379, which downregulate the TGF-β-induced expression of IL-11. Additionally, miR-124 negatively regulates IL-11 expression in vitro and in vivo, representing a key pathogenetic process in breast cancer metastasis [76]. A key mechanism in the induction of IL-11 is represented by the JAK1/STAT3 (Janus kinase/signal transducer and activator of transcription protein) pathway. Patients with breast cancer bone metastases showed higher levels of serum IL-11, tumor tissue IL-11 messenger RNA (mRNA), and IL-11 immunohistochemical staining in comparison with patients with primary breast cancer only, associated with a higher expression of p-p38, p-c-JUN, and p-STAT3, suggesting that IL-11 plays an important role in regulating bone metastasis in breast cancer, and that its level in serum may be used to assess the risk of bone metastasis from primary breast cancer [77]. Liang et al. also found that IL-11 is capable of inducing osteoclastogenesis by activating the JAK1/STAT3 signaling pathway, which induces the expression of c-Myc, a necessary factor required for osteoclastogenesis, independent of RANKL [32]. This represents a controversial issue, as McCoy and colleagues reported that IL-11 promotes RANKL-induced osteoclast (OC) differentiation [78]. A different signaling pathway can be involved, as the synthesis of IL-11 by breast cancer cells is induced by LPA through a specific PKC delta subtype, in the enhancement of breast cancer cell-mediated OC development [33]. Finally, Irawam et al. described a direct significant association between the expression of IL-11-RA and bone metastasis incidence in patients with advanced breast cancer [79].

#### 3.2.7. IL-12

One paper evaluated the in vitro synthesis of the anti-inflammatory cytokine IL-12, reporting that it is increased by the treatment of 4T1 mouse breast cancer cells with endothelin-1 [47].

#### 3.2.8. IL-15

Bottos et al. showed that the JAK/STAT pathway is activated in breast cancer bone metastasis. In fact, the blocking of the JAK/STAT pathway with JAK inhibitors (JAKi) increased the metastatic burden. The treatment of tumor-bearing mice with IL-15, a cytokine crucial for natural killer (NK), NK and memory (m) CD8+ (cluster of differentiation 8) T-cell function and homeostasis, in addition to JAKi, caused an increase in NK cell population and a reduction in metastases, preventing the JAKi-mediated increase in metastases [80].

#### 3.2.9. IL-17

Some papers focused on IL-17, a pro-inflammatory cytokine produced by breast cancer cells, hBMSCs (human bone marrow stem cells), and T-cells. Jewell et al. reported a decrease in the synthesis of IL-17 in a 4T1 mouse metastasis model upon in vivo treatment with an endothelin-1 antagonist, associated with a reduction in tumor mass and bone metastasis [47]. Goldstein evaluated the role of hBMSCs in the process of bone metastases and found that IL-17B can drive migration and metastasis. The in vitro treatment of breast cancer cells with hBMSC-derived IL-17B increased the migration of metastatic breast cancer cells, with no effect on non-metastatic breast cancer cells (MCF-7). An increase in the expression of IL-17B receptor was also observed in mouse bone metastases when compared to primary mammary tumor, as confirmed by the in vitro study in which the over-expression of IL-17BR in breast cancer cells caused an increase in migration [81]. Finally, Monteiro et al. showed that T-cells establish a pre-metastatic niche and that metastatic breast cancer cells induce the synthesis of osteclastogenic cytokines such as IL-17F and RANKL by CD4+ T-cells [40].

#### 3.2.10. IL-18

In the work by Nayir et al., the serum levels of pro-inframammary IL-18, a member of the IL-1 superfamily, were compared among breast cancer women with or without bone metastases and healthy controls, showing a decrease in IL-18 levels in response to decreased skeletal tumor burden [82].

#### 3.2.11. IL-20

One paper evaluated the function of IL-20, a cytokine with a pro-inflammatory effect structurally related to the IL-10 family, and high expression levels of IL-20 were found in primary breast tumor tissue and bone-metastatic tissue [83]. IL-20 expression in breast cancer tissue was associated with a higher mitotic rate, advanced tumor stage, and bone metastasis. IL-20 determines an increase in breast cancer cell growth, migration, and protease expression in vitro. In a mouse model of bone metastases, treatment with anti-IL-20 mAb 7E was able to reduce tumor growth, bone colonization, and osteolysis (see in Table 1) [83].

### 3.3. Findings from Clinical Trials

The 11 clinical trials included in the review studied six ILs, i.e., IL-1, IL-2, IL-3, IL-7, IL-12, and anti-IL-6R (Table 2). The most studied ILs were IL-2 (*n* = 4) and IL-12 (*n* = 3). 

IL-2 and IL-2 variants were used alone or in association with monoclonal antibodies, drugs to treat low blood neutrophils, and proteins for the evaluation of safety, tolerability, toxicity, pharmacokinetics, pharmacodynamics, and preliminary antitumor activity in treating patients with metastatic breast cancer that did not respond to previous therapy. 

In women with metastatic breast cancer, IL-12 was studied to evaluate the efficacy of high-dose chemotherapy and peripheral stem-cell transplantation or to determine its activity as defined by the percentage of patients who did not progress after six months of therapy. IL-12 may eradicate tumor cells by blocking blood flow to the tumor and by soliciting patient white blood cells to destroy breast cancer cells. An additional trial on IL-12 evaluated the toxicity and maximum tolerated dose of intratumoral injection of the adenovirus-mediated human IL-12 gene in women with metastatic breast cancer. 

Trials on the administration of IL-1, IL-3, anti-IL-6R, and IL-7 principally evaluated the toxicity, in terms of maximal tolerated dose of these ILs in metastatic breast cancer patients. However, it is important to underline that, although the majority of the trials were completed (8/11) no study results were posted in the clinical registries. The results of these clinical trials are awaited with interest because they may provide valuable information on the use of these ILs alone or in combination with other therapeutic regimes to treat patients with breast cancer bone metastasis.

## 4. Discussion

In this systematic review, we presented an overview on ILs that have an already known or putative role, function, and mechanism of action in breast cancer cells homing to bone and that were used or are actually used in therapeutic regimes for breast cancer bone metastasis management. 

The reviewed preclinical studies identified several ILs linked to breast cancer metastases to bone, i.e., IL-1β, IL-2, IL-6, IL-8, IL-10, IL-11, IL-15, IL-17, IL-18, and IL-20. Of them, IL-6, IL-8, IL-1β, and IL-11 were the ILs that showed a more critical bone tropic role. These ILs support the vicious cycle of breast cancer bone metastasis and promote bone metastasis when over-expressed or downregulated. However, the review also recognized proof for additional and potential roles and mechanisms of action of these ILs. IL-1β seems to be stimulated by OPG that acts in invasion and promotion, in the regulation of IL-11 and IL-8 production from TGF-β signaling pathways, for specific miRNAs that control TGF-β-induced IL-11 expression and for a series of mechanisms of action and roles related to IL-6 (e.g., STAT3 and its downstream effectors, Jagged1, calcium-sensing receptor, and IL6/Notch3 signaling). Most of these preclinical studies were in vitro and, in order to evaluate the abovementioned roles, functions, and mechanisms of ILs in breast cancer bone metastases pathogenesis and therapy, used different approaches. These approaches can be categorized as monoculture, co-culture, and two-dimensional (2D) and 3D cultures. This high number of in vitro studies is probably due to the fact that in vitro models are essential for high-performance assay execution. While in vivo models better mimic the native bone microenvironment in comparison to in vitro models, they frequently do not consent a methodical analysis of individual stimuli. Thus, in vitro models were probably favorable in the study of ILs in breast cancer bone metastasis, since they permitted evaluating signaling pathways, specific roles, and mechanisms of ILs critical for the metastatic colonization of bone. In this review, the analyzed in vivo studies evaluated specific IL level/expression, and, above all, the IL-based treatments after breast cancer cell injection (i.e., into blood circulation, intracardiac, intraosseous). Only small animal models were used in these studies. Several reports studied human breast cancer cell metastasis in a non-human host, using immunodeficient mice, but they did not have an immune response that could be of key importance for bone metastasis. On the other hand, some other studies used immunocompetent mice with immune response factors, but murine breast tumors basically differ from human ones. Another important issue in the preclinical studies examined in this review concerns the cell lines used, both in the in vitro and the in vivo studies that were not always completely able to mimic the intricate scenario of the clinical breast cancer bone metastasis. In detail, in the evaluated studies, the most used cell lines were human MDA-MB-231 and murine 4T1 breast cancer cells. These two cell lines are estrogen receptor-negative (ER−), although bone metastases develop more frequently from ER+ breast cancers. However, in the majority, but not all, of the studies, these cells were compared to ER+ breast cancer cells lines, such as T47D, MCF-7, ZR-75-1, and BT-474, among others. Despite many times neglected, these factors should be considered when interpreting research findings because the possibility to use ER+ breast cancer cells allows better modeling the natural processes of breast cancer metastasis to bone. Nine in vitro studies also used patient peripheral venous blood and/or tumor tissue samples. Half of these studies [49,51,77,79] reported detailed demographic and clinical–pathological information such as age of primary diagnosis of breast cancer and bone metastasis, primary breast cancer histology, hormone receptor (estrogen, progesterone) and HER2 condition, breast cancer molecular subtype, sites of metastases at breast cancer presentation, if metastatic, or at time of recurrence, date and site of disease recurrence, date of bone progression, and date of skeletal-related events (SREs), while the other half reported less or no information on derived patient fluids or tissues. However, all the studies reported the patient number and the median age, and almost all (5/8) had a control group (healthy patients or breast cancer patients without bone metastases). The presence of these clinical–pathological variables allows a more complete and accurate association between the expression of specific biomarkers (ER, Progesterone Receptor, Human Epidermal Growth Factor Receptor 2-HER2-, molecular subtypes) and ILs with breast cancer bone metastasis incidence.

Although most of the preclinical studies identified specific key ILs which promote breast cancer metastases to bone, which have a pro-metastatic effects (IL-6, IL-8, IL-1β, IL-11), and whose inhibition also shows potential preclinical therapeutic effects, the ongoing clinical trials seemed to focus principally on ILs that have anti-metastatic effects and a potential to generate a localized and systemic antitumor response. In fact, only one trial evaluated the dose-limiting toxicity of the anti-IL-6R monoclonal antibody tocilizumab in combination with trastuzumab and pertuzumab in subjects with metastatic HER2-positive breast cancer. Most of the trials focused on IL-2 and IL-12, used alone or associated with other drugs, to eradicate tumor cells by blocking blood flow to the tumor and by soliciting patient white blood cells to destroy breast cancer cells. IL-2 was characterized as a T-cell growth factor, which stimulates a broad range of effector cells, including T- and B-lymphocytes, monocytes, macrophages, and NK cells, while IL-12 causes the production of IFN-γ (interferon – γ), promotes the differentiation of helper T-cells (Th1), and establishes a connection between adoptive immunity and innate resistance. Our results show that the few preclinical literature data dealt with IL-2 or IL-12 as a response to therapies, rather than the evaluation of their contribution to the antitumor effect. Additionally, a low number of preclinical data were also present prior to 2008 and demonstrated that IL-2 and IL-12 have potent antitumor and antimetastatic activities in several murine tumor models through an immune-mediated, T-cell-dependent mechanism [84]. The trials focused on the immunotherapy principle that works to augment the cellular immune responses toward differentiation, thus achieving IL concentrations adequate to incite effector cells to induce antitumor responses in the tumor microenvironment. Despite the importance that these trials could have for the scientific community, the trial design or the information reported on the clinical registries was almost always not well structured, with the majority of the trials without randomization and with an inadequate or unclear concealment of treatment allocation. Most importantly, none of these trials posted the results and, unfortunately, without accessible and usable reports, the trials fail to help patients and their clinicians. 

However, despite the limits related to the clinical trials, the main emergent question from this IL overview is as follows: “Why are preclinical studies predominantly focused on pro-inflammatory ILs that have a pro-metastatic effect, while clinical trials are mainly oriented toward anti-inflammatory IL-based therapy that has an anti-metastatic effect?” The most correct answer could be found in popular wisdom that “most things in life are a double-edged sword”. However, this is not a scientifically correct answer, but it actually holds some truth. ILs represent a considerable number of soluble factors, and they are considered as key targets in anti-cancer immunotherapy. This review showed that a large number of pro-inflammatory ILs (IL-1, IL-6, IL-8, IL-11, and others) generated by host immune cells and/or tumor cells are linked with tumor aggressiveness, as confirmed by numerous preclinical studies. On the contrary, anti-inflammatory ILs (IL-2, IL-12, IL-10, and others) frequently activate antitumor immunity, thus interfering with tumor growth. These demonstrations could offer a promising therapeutic approach [85]. Although many IL functions are already well known, others still remain to be better evaluated, while others are yet to be discovered; thus, the understanding of the basic biological mechanisms of this complex immune microenvironment and the recognition of the effector functions should be augmented and potentiated to effectively target and destroy breast cancer bone metastasis.

## 5. Conclusions and Implications for Future Research

Bone is the preferred site for breast cancer metastasis. In vitro studies suggest that some pro-inflammatory ILs firmly correlate with augmented breast cancer cell aggressiveness. Even though in vivo reports are in their primitive stages, those performed up to now confirmed these findings. In particular, one review highlighted that some IL signaling is associated with breast cancer bone metastasis, and the inhibition of these ILs leads to a reduction in bone metastases. Conversely, other clinical trials focused on anti-inflammatory IL therapies that should correlate with reduced aggressiveness of breast cancer cell lines through the killing of tumor cells, by stopping the blood flow to the tumor and stimulating patient white blood cells to eradicate breast cancer cells. However, no conclusion can be finalized regarding clinical trials since none of them reported results on the safety, tolerability, toxicity, pharmacokinetics, pharmacodynamics, and preliminary antitumor activity of these therapies.

Further preclinical studies are specifically necessary to augment the understanding of cellular and molecular relations and signaling pathways, both up- and downstream of ILs recognized in this review, which have or could have critical roles in breast cancer metastases to bone. Additionally, clinical trials with detailed specifications on randomization, concealment of treatment allocation, blinding, co-interventions, compliance to the interventions and timing of outcome assessment for intervention, and posted results are mandatory, since they may be able to identify supplementary essential factors and/or therapies for breast cancer metastases to bone.

Based on the preclinical results and on the initial clinical results, the translation potential of IL-based therapies could represent a novel therapeutic approach for breast cancer patients at risk of progressing to bone metastasis. However, despite these preliminary results, to date, IL-based therapies still require more extensive analyses to confirm and extend their use through the design of new and advanced preclinical studies and prospective randomized trials. 

## Figures and Tables

**Table 1:** Preclinical studies on interleukins (ILs) and breast cancer bone metastases.

References	Interleukin(s) Identified	Interleukin Source	Aims	Experimental Approaches
[68]	IL-8	Breast cancer cells	Evaluate the influence of the expression of IL-8 on bone resorption in osteolytic breast cancer cell lines.	In vitro MDA-MB-231, MDA-231B, T-47D, ZR-75-1 culture.
[54]	IL-6 IL-8	Osteoblasts	Evaluate osteoblasts inflammatory stress response (IL-6, IL-8) in presence of metastatic breast cancer cells.	In vitro hFOB 1.19 co-culture with MDA-MB-231 CM; hFOB 1.19 and MDA-MB-231 co-culture.
[18]	IL-1β	Osteoblasts	Evaluate if OPG production by breast cancer cells, pretreated or not with IL-1β, correlates with bone colonization.	In vitro hFOB 1.19 and 435/BRMS1, 231/BRMS1, 231/K and MDA-MB-435 derived from the pleural effusion of a female patient with an infiltrating ductal carcinoma or their CM (pretreated or not with IL-1β) co-culture.
[25]	IL-6	Breast cancer cells	Evaluate fibroblasts isolated from breast cancer metastasis and the link with tumor growth rates.	In vivo and in vitro MCF-7 and BT474 co-cultured with Ped300 and P162 fibroblast cells CM. MCF-7 cells injected s.c. (± fibroblasts) in the dorsal area of BALB/c nude mice
[20]	IL-8	Osteoblasts	Evaluate osteoblast-derived TGF association with osteolytic bone diseases and ILs regulation.	In vitro MDA-231 and MDA-435 co-cultured with MG-63 CM.
[55]	IL-6	Osteoblasts	Evaluate colonization and degradation of osteoblastic tissue by breast cancer cells.	In vitro MDA-MB-231 CM co-cultured with osteoblast tissue.
[52]	IL-6	Peripheral venous blood	Identify clinical significance of serum IL-6 and its correlation with Cyst C in patients.	In vitro Peripheral venous blood from 25 healthy donors,10 patients with age-related osteoporosis, 16 patients with localized breast cancer, 10 patients with breast cancer bone metastases.
[34]	IL-8 IL-6	Breast cancer cells	Evaluate the activity of S1P and LPA on breast cancer cells to stimulate osteoclasts.	In vitro MDA-MB-231/BO2 (MDA- BO2) CM untreated or treated with S1P and LPA. Mice bone marrow cells cultured with MDA-BO2 CM treated with S1P and LPA.
[31]	IL-8	Breast cancer cells	Evaluate the role of Syndecan-1 in osteoclastogenesis.	In vitro Human PBMC: mCSF present in all groups; RANKL or IL-8 used as positive controls. Human PBMCs that received mCSF cultured with CM from MDA MET-derived cell lines HPSE-high, HPSE-low, M225, and M343. For same cultures, Syndecan-1 immunodepleted from CM using monoclonal antibody BB4 and protein-G sepharose beads.
[60]	IL-6	Osteoblasts	Determine the localization of osteoblast-derived IL-6.	In vivo MDA-MB-231 expressing GFP variant injected into the left cardiac ventricle of athymic mice.
[56]	IL-6 IL-8	Osteoblasts	Identify key cytokines expressed by osteoblasts in metastatic breast cancer cells.	In vitro and in vivo MC3T3-E1 co-cultured with MDA-MB-231 variants CM; MDA-MB-231 variants injected into the left cardiac ventricle of athymic mice.
[47]	IL-1α IL-17 IL-12	Breast cancer cells	Evaluate if limiting ET-1 expression and activity (blocking ETAR and ETBR) reduced breast cancer growth and development of tumor infiltration in bone.	In vitro and vivo 4T1 cells injected in C57Bl/6× Balb/C male mice and treated with vehicle) or with ETAR/ETBR dual selective antagonist. 4T1 cells CM treated with ET-1.
[57]	IL-6	Osteoblasts	Evaluate the dynamic interaction between breast cancer cells and osteoblastic tissue.	In vitro MDA-MB-231 and MC3T3-E1 co-culture; MDA-MB-231 inoculated into bioreactor containing osteoblastic tissue.
[81]	IL-17B	Bone marrow mesenchymal cells	Evaluate the role of hBMSC in metastatic breast cancer cells.	In vitro and in vivo Migration assay of hBMSC to human BCC (SUM 1315, SUM 1315-BP2, MDA-MB-231 and MCF7), wild type (wt) or over-expressed for IL-17 br, or treated with IL-17B. In vivo metastasis model of hBMSC and breast cancer cells(SUM 1315, SUM 1315-BP2, MDA-MB-231 and MCF7) wt or over-expressed for IL-17 br, co-injected in mice mammary fat pad.
[58]	IL-6	Osteoblasts	Identify if Notch-dependent signaling proteins secreted by osteoblasts may stimulate tumor growth.	In vitro MC3T3-E1 and JAG1 OE breast cancer cells co-culture.
[74]	IL-11	Breast cancer cells	Determine how Runx2 mediates the ability of metastatic breast cancer cells to modulate the activity of bone cells.	In vitro MC3T3-E1 co-cultured with MDA-MB-231 CM or sclerostin-free CM; Mouse BMSCs co-cultured with MDA-MB-231 CM or sclerostin-free CM.
[21]	IL-8 IL-11	Breast cancer cells	Delineate TGF-β signaling pathways in the production of IL-8 and IL-11 in breast cancer cells with different bone metastatic potential.	In vitro MDA-MB-231, MDA-MB-468, and MCF-7 cultured with TGF-β. MDA-MB-231 treated with SMAD4 RNAis or MAPK14 RNAis and low GC content negative control RNAi and treated with TGFβ-1.
[30]	IL-1β IL-8	Breast cancer cells	Identify if TAK1 (TGF-β-activated protein kinase 1) effectors contribute to bone metastatic potential of breast cancer cells.	In vitro Suppression of TAK1 signaling in MDA-MB-231 cells by expressing the dominant-negative TAK1-K63W mutant allele or by siRNA. Control: empty-vector. CM from control and dn-TAK1-expressing MDA-MB-231 cells treated with TGF-β1 or/and IL1-β
[61]	IL-6		Evaluate the clinical relevance of gene expression signatures induced by heterotypic interaction of breast cancer cells and osteoblasts.	In vitro Co-culture of HMECs; MCF-7, T47D, MDA-MB-231, SKBR-3 and Hs578T co-culture with primary human osteoblasts.
[22]	IL-11	Breast cancer cells	Identify and characterize miRNAs that regulate the TGF-β induction of IL-11 in metastatic breast cancer cells.	In vitro MDA-MB-231(SA) transfected with precursors or miRNA inhibitors and cultured with TGF-β.
[23]	IL-11	Breast cancer cells	Evaluate gene for TGF-β-induced IL-11 production in highly bone metastatic MDA-MB-231(SA) by a cell-based siRNA screen.	In vitro MDA-MB-231(SA) transfected with siRNAs cultured with heparin, fragmin, or K5-NSOS and TGF-β.
[24]	IL-6 IL-8	Breast cancer cells	Evaluate an unbiased genome-wide miRNA screen to identify miRNAs modulating NF-κB signaling in metastatic breast cancer cells.	In vitro MDA-MB-231 transfected with miR-373 or miR-520c and treated with TNF-α or TGF-β.
[83]	IL-20	Tumor tissue Breast cancer cells	Evaluate the function of IL-20 in tumor growth, metastasis, and clinical outcome.	In vitro and in vivo IL-20, IL-20 receptors, and anti–IL-20 mAb 7E immunohistochemical staining of tumor tissue samples with primary intraductal carcinoma of the breast, with breast cancer, with tumorous and non-tumorous breast tissue with breast cancer bone metastases, and breast cancer cell lines (MDA-MB231 and MCF-7); Intracardiac and intratibial injection of 4T1-Luc in BALB/c mice.
[49]	IL-6	Serum	Determine serum level of IL-6 protein clinical utility in patients with breast cancer bone metastasis.	In vitro Blood samples from 164 patients with stage I–III breast cancer.
[75]	IL-11	Breast cancer cells	Evaluate the effect of TGFβ-1 on IL-11 production.	In vitro 4T1 cells exposed to different doses TGFβ-1.
[35]	IL-6 (Oncostatin M-OSM)	Breast cancer cells	Evaluate the role of OSM in the formation of bone metastases.	In vitro and in vivo 4T1.2-OSM (OSM expression knocked down using shRNA) and 4T1.2-LacZ (control) injected into the mammary fat pad or intra-tibial in Balb/c mice. RAW 264.7 co-cultured with 4T1.2.
[40]	IL-17F IL-1β IL-6	Breast cancer cells T-cells	Evaluate how the pre-metastatic niches arise in the bone tissue.	In vitro and in vivo Inoculation of non- metastatic 67 NR or metastatic 4T1 BCC in the mammary fat pad of BALB/c mice. T-cells from 4T1 tumor-bearing mice KD for RANKL and IL-17F, and transferred into Balb/c nude mice.
[77]	IL-11	Serum Tumor tissue	Evaluate the relationship between IL-11 and breast cancer bone metastasis in patients.	In vitro Blood samples from patients with bone metastasis, with primary cancer and healthy patients; formalin-fixed, paraffin-embedded tumor tissue (from patients with bone metastasis and from patients with primary cancer); fresh tumor tissue from patients (with primary cancer and with breast cancer metastasis).
[78]	IL-11	Breast cancer cells	Investigate the role of IL-11 in metastasis-induced osteolysis.	In vitro Bone marrow cells from femur and tibia of C56BL/6 mice co-cultured with MDA-MB-231 CM; IL-11 neutralizing antibody added to bone marrow in 20% MDA-MB-231 CM. Bone marrow cultured with IL-11 to generate osteoclasts from IL-11-dependant precursors.
[53]	IL-6 IL-8	Cancer stem cells	Investigate the ability of non-metastatic human breast cancer stem cells to metastasize to bone.	In vivo Primary CD44^+^ CD24^−^ breast CSCs-like transduced by luciferase-lentiviral vector injected s.c. and intra-cardiac in immunodeficient mice carrying s.c. human bone implants. Mammospheres derived from patient tumor specimens.
[59]	IL-6	Osteoblasts	Determine whether 3D mineralizing tissue would be a bone surrogate for studying the early stages of breast cancer colonization to bone.	In vitro MC3T3-E1 (grown for 60 days in bioreactor to form a 3D collagenous osteoblastic tissue), pre-osteoclasts (obtained from bone marrow cells harvested from 6- to 9-week-old GFP mice) and MDA-MB-231-GFP.
[64]	IL-6	Breast cancer cells	Investigate the effects of RANKL on cancer cells and the role of tumor-derived IL-6 within the bone microenvironment.	In vitro and in vivo. Normal or modified (silenced IL-6 and RANK via a lentiviral-based system) MDA-MB-231 injected intra-tibial or s.c. in e BALB/c nu/nu mice.
[39]	IL-1β	Breast cancer cells	Identify changes in gene and protein expression associated with bone-homing or colonization, developing a novel bone-seeking clone of MDA-MB-231 cells.	In vitro and in vivo Intra-cardiac injection of MDA-P cells in female Balb/c nude mice. Cells that formed colonies in mouse tibiae were extracted and pooled before culture in vitro. Following 1 week of culture, cells were re-injected into the mice left cardiac ventricle or tail vein. Procedure repeated 7 times until tumor growth in mouse long bones was detected following intravenous injection (MDA-IV).
[62]	IL-6	Breast cancer cells	Assess the effect of soluble mediators produced by breast cancer cells on human osteoclast maturation.	In vitro Differentiation toward osteoclasts (from healthy volunteer) induced by α-MEM supplemented with MCS-F and RANKL or by α-MEM supplemented with 10% MDA-MB-231 (CM).
[69]	IL-8	Plasma	Identify IL-8 association with increased bone resorption and breast cancer bone metastasis.	In vitro and in vivo Plasma from patients with or without bone metastasis from breast cancer; MDA-MET injected into the tibia of nude mice and 7 days later treated daily with a neutralizing IL-8 monoclonal antibody.
[48]	IL-2 IL-6	Blood	Evaluate the possible relationship between RPT efficacy and cytokines levels.	In vitro Blood samples from patients with bone metastases.
[72]	IL-8	Osteoblasts	Test the cell migration stimulated by OCM.	In vitro MCF-7 cultured with hFOB1.19 human osteoblasts CM.
[44]	IL-1β	Osteoblasts	Test if bone remodeling cytokines could stimulate dormant cells to grow.	In vitro MDA-MB-231BRMS1 and MC3T3-E1 co-cultured in a long term 3D system.
[71]	IL-8	Osteoblasts	Evaluate the link between osteoblasts and breast cancer cells in healthy and osteoporotic conditions.	In vitro Osteoblasts isolated from trabecular bone of iliac crest of SHAM and OVX rats co-cultured with MRMT-1 rat breast cancer cells CM.
[41]	IL-1β	Breast cancer cells	Identify parameters of human bone tissue associated with breast cancer cell osteotropism and colonization.	In vitro MDA-MB-231-fLuc-EGFP and MCF-7-fLuc-EGFP co-cultured with cancellous bone tissue fragments from patients.
[26]	IL-6	Breast cancer cells	Evaluate the association between HT and breast cancer bone metastases.	In vitro and in vivo Tumor-derived cancer cells from xenografts and primary human ductal carcinoma tissues. Xenografts from tumorigenic MCF7 clones administered with tamoxifen citrate implants, fulvestrant and tocilizumab. Cancer cells injected bilaterally in the mammary fat pads of NOD/SCID mice.
[27]	IL-6	Breast cancer cells	Evaluate the ability of CaSR to play a chemotactic and pro-angiogenic role in MDA-MB-231 breast cancer cells by cytokines secretion.	In vitro MDA-MB-231 and MCF-12A CM stimulated with different Ca^2+^ concentrations.
[42]	IL-1β	Breast cancer cells	Generate a clinically relevant model for the study of breast cancer tumor cell-bone interactions.	In vitro and in vivo Human bone discs from patients and DiD labeled MDA-MB-231-luc2 co-culture; tumor-cell bearing bone discs implanted s.c. into the flanks of NOD SCID nude mice; MDA-MB-231 luc2 injected into the hind mammary fat pads of NOD SCID nude mice.
[70]	IL-8	Breast cancer cells	Investigate breast cancer cell IL-8 expression in response to systematic changes of HA.	In vitro MDA-MB-231, MCF-7, MCF10A, MCF10AT1, and MCFDCIS breast cell seeded onto mineral coatings Poly (d,l-lactide-*co*-glycolide) PLG.
[66]	IL-6	Osteoblasts	Study reactive senescent osteoblasts and evaluate if they increased breast cancer colonization to bone.	In vitro and in vivo NT2.5 and BMMs co-cultured with senescent or non-senescent FASST osteoblasts CM + IL-6 neutralization antibody or IgG control; senescent vs. control osteoblasts co-cultured with mice BMMs. NT2.5luc injected into the left cardiac ventricle of FASST or littermate control mice that received either anti-murine IL-6 antibody or anti-murine IgG2a antibody.
[67]	IL-6 IL-8	Breast cancer cells	Evaluate the effect of LMW-PTP slow isoform in tumor cell induced osteoclastogenesis	In vitro MDA-MB-435 KD for LMW-PTP co-cultured with Raw 264.7.
[82]	IL-18	Serum	Compare serum IL-18 levels in breast cancer patients with and without bone metastases	In vitro Blood samples from female breast cancer patients with or without bone metastases and from healthy subjects.
[79]	IL-11RA	Tumor tissue	Analyze expression of IL11-RA in advanced breast cancer patients with or without bone metastasis.	In vitro Human tissue samples from breast cancer patients with or without bone metastases.
[80]	IL-15	Tumor tissue Breast cancer cells	Study JAK/STAT pathway in the bone metastasis from breast cancer.	In vitro and in vivo Breast cancer and bone metastases biopsies from patients. Intra-mammary and intra-cardiac injection of MDA-MB-231 scp1833 and EO771 cells in nude mice.
[45]	IL-1	Breast cancer cells	Investigate the blocking IL-1R signaling with the clinically licensed antagonist, i.e., anakinra.	In vitro and in vivo Co-culture of MDA-MB-231-IV (eGFP-expressing bone-homing derivative of MDA-MB-231) or MCF7 with HS5 human bone marrow cells. Mice pretreated with anakinra or placebo starting 3 days before injection of MCF7 or MDAMB- 231-IV cells s.c. or i.v. via the lateral tail vein; MDA-MB-231-IV cells injected s.c. or i.v. 7 days prior to commencement of anakinra treatment. Mice injected with MCF7 cells were supplemented with B-estradiol.
[50]	IL-6	Serum	Evaluate serum levels of IL-6 in breast cancer patients with or without metastasis.	In vitro Blood samples from patients with breast cancer, with metastatic breast cancer and from healthy subjects.
[43]	IL-6 IL-1β IL-10 IL-8	Breast cancer cells	Evaluate the relationship between osteoporosis and breast cancer-derived bone metastases in a humanized 3D model.	In vitro Bone tissue samples from healthy and osteoporotic patients co-cultured with MCF-7.
[36]	IL-6	Breast cancer cells	Identify tumor-secreted cytokines regulated by the ABL kinases that promote breast cancer metastasis to bone.	In vitro CM of 1833 (bone metastasis sub-line derived from MDA-MB-231 breast cancer cells) cells transduced with control shRNA (Scr) or shRNAs against ABL1 and ABL2 (shAA). The Cancer Genome Atlas dataset analyses.
[37]	IL-8	Osteoblasts	Evaluate Sema4D-mediated induction of IL-8 and LIX/CXCL5.	In vitro and in vivo HOB and MC3T3 CM cultured with Sema4D. HOB cultured with CM by MDA-MB-231 or cells silenced for Sema4D, with and without *Clostridium botulinum* toxin C3. RAW264.7 cultured with RANKL, IL-8, or CM by HOB treated with sSema4D or empty vector transfected controls and anti-IL-8 antibody. MDA-MB-231 infected ex vivo with control virus or lentivirus coding for Sema4D shRNA injected into the left cardiac ventricle of nude mice.
[73]	IL-8	Breast cancer cells	Investigate the interaction between breast cancer cell and osteoblasts in a 3D printed bone matrices.	In vitro hFOB and MDA-MB-231 cells co-cultured on bone matrix.
[51]	IL-6	Blood	Assess the correlation between sYB-1 and serum IL-6 in patients with breast cancer bone metastasis.	In vitro Peripheral blood from patients with breast cancer bone metastasis.
[19]	IL-1β	Breast cancer cells Macrophages	Evaluate the link between OPG, macrophages, and IL-1β.	In vitro MDA-MB-231, MDAMB-436, BT549, SKBR3, ZR75-1, HCC1954 human breast cancer cells transfected with OPG Stealth RNA siRNA; Breast cancer cells and THP-1 monocyte cells (previously treated with PMA to induce macrophage differentiation) co-culture.
[65]	IL-6	Breast cancer cells	Study the effects of IL-6 receptor on breast cancer aggressiveness and bone metastases.	In vitro and in vivo MDA-MB-231 cultured in presence or absence of tocilizumab (anti-human IL-6 receptor (IL-6R). Intracardiac injection of MDA-MB-231 in BALB/c nu/nu mice.
[28]	IL-6	Breast cancer cells	Evaluate the inhibition of IL-6 signaling using a molecule antagonist, TB-2-081, on bone integrity, tumor progression, and pain.	In vitro and in vivo MAT B III cells pretreated with either vehicle or TB-2-081. Intratibial injection of MAT B III in Fisher F344/NhSD rats and treatment with either vehicle or TB-2-08. Osteoblasts harvested from 24- to 48-hour-old neonatal Fischer F344/NhSD pups exposed to vehicle or TB-2-081 pretreatment before challenge of IL-6 recombinant protein.
[76]	IL-11	Breast cancer cells	Investigate miR-124/IL-11 in the prognosis of breast cancer patients with bone metastasis.	In vitro and in vivo MDA-MB-231 cells transfected with miR-124 mimic (miR-124) and MCF7 cells transfected with miR-124 inhibitor (in-miR-124). MCF7 stably expressing miR-124 inhibitor or NC inoculated into left ventricle of nude mice.
[38]	IL-6 (Oncostatin M-OSM)	Macrophage	Evaluate OSM induced macrophage M2 polarization during breast cancer bone metastasis.	In vitro and in vivo MDA-MB-231 CM cultured under hypoxia or normoxia with THP-1 cells differentiated to macrophages. BALB/c mice s.c. injected with 2 doses of 4T1 cells in the T4 of mammary fat pad. Mouse PBMCs transfected with the siRNA targeted against Rictor and quantum dots (Q-Dots) before being reintroduced in mice. Macrophages isolated from tumor tissue.
[33]	IL-8 IL-11	Breast cancer cells	Evaluate the role of LPA in the regulation of osteoclastogenic cytokines from breast cancer cells.	In vitro MDA-MB-231 CM and MDA-MB-468 containing LPA co-cultured with RAW264.7 cells
[32]	IL-11	Breast cancer cells	Evaluate the role of IL-11 in bone metastasis from breast cancer.	In vitro and in vivo MCF-7, MDA-MB-231 and BoM-1833 CMs (infected with UBI-MCS-EGFP-SV40-Firefly-Luciferase-IRES Puromycin) co-cultured with BMMs (transfected with siRNA against mouse STAT3, c-Myc, and control si-RNA). Block of osteolytic factors in BoM-1833 cells CM through monoclonal antibodies against VEGF, PTHrP, IL-11, and CTGF. MCF-7, MDA-MB-231, and BoM-1833 injected into femur of BALB/c-nu/nu nude mice. STAT3 inhibitor AG-490 i.v. injected every 2 days for 3 weeks.
[63]	IL-6	Breast cancer cells	Evaluate TNF-α and IL-6 in the pathophysiology of pain syndrome in breast cancer bone metastasis.	In vivo Tumor section, obtained from Sprague-Dawley rats injected s.c. with CRL-1666 breast cancer cells, implanted in L6 vertebra bone defect.
[46]	IL-1β	Breast cancer cells	Identify how tumor cell derived IL-1β drives breast cancer progression and bone metastasis; effect of targeting the IL-1β pathway using the anti-human IL-1β antibody, canakinumab, and IL-1R antagonist, anakinra.	In vitro and in vivo Patient tissues with stage II/III breast cancer. MDA-MB-231, MCF-7, and T47D cells (transfected to over-express IL-1β or IL-1R1) co-cultured with OB1. MDA-MB-231 or T47D cells co-cultured with human bone discs. Human bone discs implanted s.c. into NOD SCID mice; after 4 weeks MDA-MB-231 Luc2-TdTomato, MCF7 Luc2, or T47D Luc2 injected into hind mammary fat pads. IL-1Ra or canakinumab s.c. in NOD SCID mice, starting 7 days after injection of tumor cells. IL-1Ra administered for 21 or 31 days or canakinumab administered as a single s.c. injection in BALB/c and C57BL/6 mice. Injection of MDA-MB-231 GFP, MDA-MB-231 IV, MDA-MB-231 IL-1B+, or MDA-MB-231 IL-1R1+ cells into tail vein of BALB/c nude mice or following intra-ductal injection of E0771 into mammary ducts of IL-1B-KO or fl/fl.
[29]	IL-8	Breast cancer cells	Evaluate the knockdown of Osx in breast cancer bone metastasis.	In vivo Transfected MDA-MB 231 cells with stable Osx knockdown or Osx over-expression, injected into the tibiae of nude mice.

Abbreviation: IL = interleukin; CM = conditioned medium; OPG = osteoprotegerin; hFOB = human fetal osteoblast; β = beta; 2D = two dimensional; 3D = three dimensional; STAT3 = signal transducer and activator of transcription 3; CA-IX = carbonic anhydrase isoenzyme IX; TGF = transforming growth factor; MAPK = mitogen-activated protein kinase; BMP-2 = bone morphogenetic protein 2; IGF-1 = insulin-like growth factor 1; AP-1 = activator protein 1; NF-κB = nuclear factor kappa-light-chain-enhancer of activated B cells; Cyst C = cystatin C; GFP = green fluorescent protein; TRAP = tartrate-resistant acid phosphatase; RANKL = receptor activator of nuclear factor kappa-Β ligand; ET-1 = endothelin-1; ETR = endothelin receptor; dH_2_O = distilled water; α = alpha; Runx2 = runt-related transcription factor 2; CBFβ = core-binding factor β; RNAis = RNA interference; GC = guanine–cytosine; HMECs = human mammary epithelial cells; K5-NSOS = k5-derived heparin-like polysaccharide; HS6ST = heparan sulfate 6 osulfotransferase; HS3ST1 = heparan sulfate glucosamine 3-*O*-sulfotransferase 1; HLGAG = heparin-like glycosaminoglycan; TNF = tumor necrosis factor; mAb = monoclonal antibody; LN = lymph nodes; CSCs = cancer stem cells; α-MEM = minimum essential medium α; M-CSF = macrophage colony-stimulating factor; ER = estrogen; RPT = radionuclide palliative therapy; OCM = osteoblast-conditioned medium; RB = receptor type B; PGE2 = prostaglandin E2; SHAM = sham-operated; OVX = ovariectomized; HT = hormonal therapy; MS = mammosphere; OXPHOS = oxidative phosphorylation; CaSR = calcium sensing receptor; HA = hydroxyapatite; PLG = poly (d,l-lactide-*co*-glycolide); mSBF = modified simulated body fluid; IgG = immunoglobulin G; BMMs = bone marrow macrophages; ROC = receiver operating characteristic; s.c. = subcutaneously; i.v. = intravenously; sYB-1 = synaptobrevin homolog 1; MMP-3 = matrix metalloproteinase-3; PMA = phorbol 12-myristate 13-acetate; IL-6R = anti-human IL-6 receptor; VEGF = vascular endothelial growth factor; NC = negative control; LPA = lysophosphatidic acid; LPARs = lysophosphatidic acid receptors; EMT = epithelial-to-mesenchymal transition; Osx = Osterix; S1P = sphingosine 1-phosphate; HPSE-low = heparanase; TAK1 = TGF-β-activated protein kinase 1; OSM = oncostatin M; OSMRβ = oncostatin M receptor β; LIFRβ = Leukemia Inhibitor Factor receptor β; LMW-PTP = low-molecular-weight protein tyrosine phosphatase; PTHrP= Parathyroid hormone-related protein; CTGF = connective tissue growth factor.

**Table 2:** Clinical trials on ILs and breast cancer bone metastases.

Trial Number and Status	Aim of the Trial	Trial Arms	Interleukin(s) Identified and Dosage	Function of Interleukin(s)	Summary of Main Trial Findings
NCT00006228 Completed	Study the effectiveness of trastuzumab + IL-2 in patients with metastatic breast cancer that did not respond to trastuzumab therapy alone.	Trastuzumab IV and aldesleukin SC.	IL-2 Trastuzumab over 30–90 min on days 1 and 8 and aldesleukin on days 2–7 and 9–21. Beginning on day 22, trastuzumab over 30 min every 14 days and aldesleukin daily on days 1–14. Treatment continues for 1 year.	IL-2 may stimulate patients white blood cells to kill breast cancer cells.	No results posted
NCT00001269 Completed	Determine the maximal tolerated dose of IL-3 given alone or sequentially with GM-CSF following FLAC CT in metastatic breast cancer patients.	IL-3 alone or sequentially with GM-CSF.	IL-3 Dosage not reported.	IL-3 and GM-CSF may ameliorate cumulative thrombocytopenia after FLAC CT.	No results posted
NCT00004893 Completed	Determine the activity of IL-12 as defined by the % of patients who did not progress after 6 months of therapy.	Arm I: Patients begin therapy no sooner than 3 and no later than 6 weeks since last CT dose and receive IL-12 SC twice a week. Patients are followed every 3 months for 1 year, and, if no progression, for 5 yrs. Arm II: Patients are observed for 6 months and, if disease progresses or not, they may receive IL-12 as in arm I. Patients are followed for toxicity until IL-12 is discontinued.	IL-12 Dosage not reported.	IL-12 may kill tumor cells by stopping blood flow to the tumor and by stimulating patients white blood cells to kill breast cancer cells.	No results posted
NCT00003412 Study completion date passed, and status not verified in more than 2 years	Study the effectiveness of IL-12 in women with metastatic breast cancer who have received high-dose CT, and peripheral stem cell transplantation.	Arms not reported.	IL-12 Dosage not reported.	IL-12 may kill tumor cells by stopping blood flow to the tumor and by stimulating patients white blood cells to kill breast cancer cells.	No results posted
NCT00849459 Completed	Determine toxicity and maximum tolerated dose of intratumoral injection of ADV-hIL12 gene in women with metastatic breast cancer.	ADV-hIL12	IL-12 Starting dose of ADV-hIL12 to 1 × 10 to the 10th power vp per patient, escalation in half-log increments up to 1 × 10 to the 13th power vp per patient, dose escalation at lower increments of 2 × 10 to a maximum of 3.0 × 10 to the 13th power vp per patient.	Placing the gene for IL-12 into breast cancer cells may help the body build an immune response to kill tumor cells.	No results posted
NCT00002616 Study completion date passed, and status not verified in more than 2 years	Study the effectiveness of IL-2 + G-CSF to stimulate cell production in patients with stage IIIB, stage IV, metastatic, or recurrent breast cancer to undergo peripheral stem-cell transplantation.	Arm I: G-CSF for priming and following stem cell transplant. Arm II: G-CSF for priming and G-CSF + IL-2 following transplant. Arm III: G-CSF + IL-2 for priming and G-CSF following transplant. Arm IV: G-CSF + IL-2 for priming and following transplant. Cohorts of 3–6 patients each treated on each arm and at escalating doses of IL-2.	IL-2 Dosage not specified.	Estimate the maximum tolerated dose of continuous infusion IL-2 combined with a dose of G-CSF to stimulate PBSC for harvest in patients with advanced breast cancer.	No results posted
NCT00001270 Completed	Define the toxicity of IL-1 administered for 7 days prior to ICE CT.	Arms not reported.	IL-1 Dosage not specified.	IL-1 toxicity.	No results posted
NCT01368107 Completed	Evaluate the impact of an immunotherapy by IL-7 on CD4 lymphopenia (CYT107), risks of severe hematological toxicity and tumor progression in metastatic breast cancer patients.	Arm I: Placebo comparator; Arm II: CYT107 SC before CT; Arm III: CYT107 during CT; Arm IV: CYT107 before and during CT.	IL-7 Arm I: Placebo before the 1st (D0, D7, D14) and during the 3rd CT cycle (D57, D64, D71); Arm II: CYT107 (10 µg/kg/week for 3 weeks) before the 1st cycle and placebo during the 3rd cycle; Arm III: placebo before the 1st cycle and CYT107 during the 3rd cycle; Arm IV: CYT107 before the 1st cycle and IL-7 (10µg/kg/week SC for 3 weeks) during the 3rd cycle.	Immunotherapy by IL-7 on CD4 lymphopenia.	No results posted
NCT02627274 Recruiting	Evaluate safety, tolerability, pharmacokinetics, pharmacodynamics, and preliminary antitumor activity of RO6874281, an immunocytokine consisting of IL-2v targeting FAP, as single agent or in combination with trastuzumab or cetuximab.	Arm I: RO6874281 monotherapy; Arm II: RO6874281 + trastuzumab; Arm III: RO6874281 + cetuximab.	IL-2v Arm I: 5 mg of RO6874281 as single agent OW; Arm II: 10 mg of RO6874281 + trastuzumab (loading dose 4 mg/kg and maintenance dose 2 mg/kg from cycle 2) OW; Arm III: 5 mg of RO6874281 + cetuximab (loading dose 400 mg/m^2^ and maintenance dose 250 mg/m^2^ from cycle 2) OW.	Not specified	Not applicable
NCT00002780 Unknown	Evaluate the toxicities of low-dose IL-2 and GM-CSF, and multiple doses of activated T-cells following PBSC transplantation in women with stage IIIB or metastatic adenocarcinoma of the breast.	Patients receive GM-CSF SC daily for 5 days prior to PBSC collection or for 2 days prior to bone marrow harvest. Following collection, patients receive high dose CT on days −6 and −3. Patients undergo PBSC transplantation on day 0. Following transplantation, patients receive multiple doses of monoclonal antibody OKT3 activated T lymphocytes IV between days 1 and 65, continuous low dose IL-2 IV over days 1–65, and GM-CSF on days 5–21.	IL-2 Dosage not specified.	Evaluate if T-cells and IL-2 combined with PBSC transplantation or bone marrow transplantation in women who have stage IIIB or metastatic breast cancer may kill more tumor cells.	No results posted
NCT03135171 Recruiting	Determine the highest dose level of anti-IL-6R (tocilizumab) that, when given in combination with trastuzumab and pertuzumab every 3 weeks in subjects with HER2-positive metastatic breast cancer, results in less than 25% incidence of dose limiting toxicity.	Trastuzumab, pertuzumab, and tocilizumab.	Anti-IL-6R Trastuzumab: all dose levels receive 8 mg/kg loading dose for cycle 1 and 6 mg/kg in subsequent cycles, every 3 weeks. Pertuzumab: dose levels 2 and 3 receive 840 mg loading dose for cycle 1 and 420 mg in subsequent cycles, every 3 weeks. Tocilizumab: 4–8 mg/kg administered IV every 3 weeks.	Safety, tolerability, and recommended phase 2 dose of tocilizumab.	No results posted

Abbreviation: IL = interleukin; IV = intravenously; SC = subcutaneously; GM-CSF = granulocyte macrophage colony-stimulating factor; FLAC = fluorouracil, leucovorin, adriamycin, cytoxan; CT = chemotherapy; ADV-hIL12 = adenovirus-mediated human interleukin 12; vp = virus particles; G-CSF = granulocyte colony-stimulating factor; PBSC = peripheral blood stem cells; ICE = ifosfamide, carboplatin, etoposide; IL-2v = interleukin-2 variant; FAP = fibroblast activation protein; HER2 = human epidermal growth factor receptor 2; OW = once weekly.
